# Correction: The odorant metabolizing enzyme UGT2A1: Immunolocalization and impact of the modulation of its activity on the olfactory response

**DOI:** 10.1371/journal.pone.0299662

**Published:** 2024-02-23

**Authors:** Fabrice Neiers, David Jarriault, Franck Menetrier, Philippe Faure, Loïc Briand, Jean-Marie Heydel

## Notice of republication

This article was republished on February 15^th^, 2024, to correct the author list and add Philippe Faure as the fourth author. Please download this article again to view the correct version. The originally published, uncorrected article and the republished, corrected articles are provided here for reference.

## Supporting information

S1 File Originally published, uncorrected article. (PDF)

S2 File Republished, corrected article. (PDF)

## References

[1] Neiers F, Jarriault D, Menetrier F, Faure P, Briand L, Heydel J-M (2021) The odorant metabolizing enzyme UGT2A1: Immunolocalization and impact of the modulation of its activity on the olfactory response. PLoS ONE 16(3): e0249029. 10.1371/journal.pone.0249029 33765098 PMC7993815

