# Supplementary figures and images for: The odorant metabolizing enzyme UGT2A1: Immunolocalization and impact of the modulation of its activity on the olfactory response
Source: PLoS One. 2021 Mar 25;16(3):e0249029. doi: 10.1371/journal.pone.0249029 (PMC7993815; doi:10.1371/journal.pone.0249029)

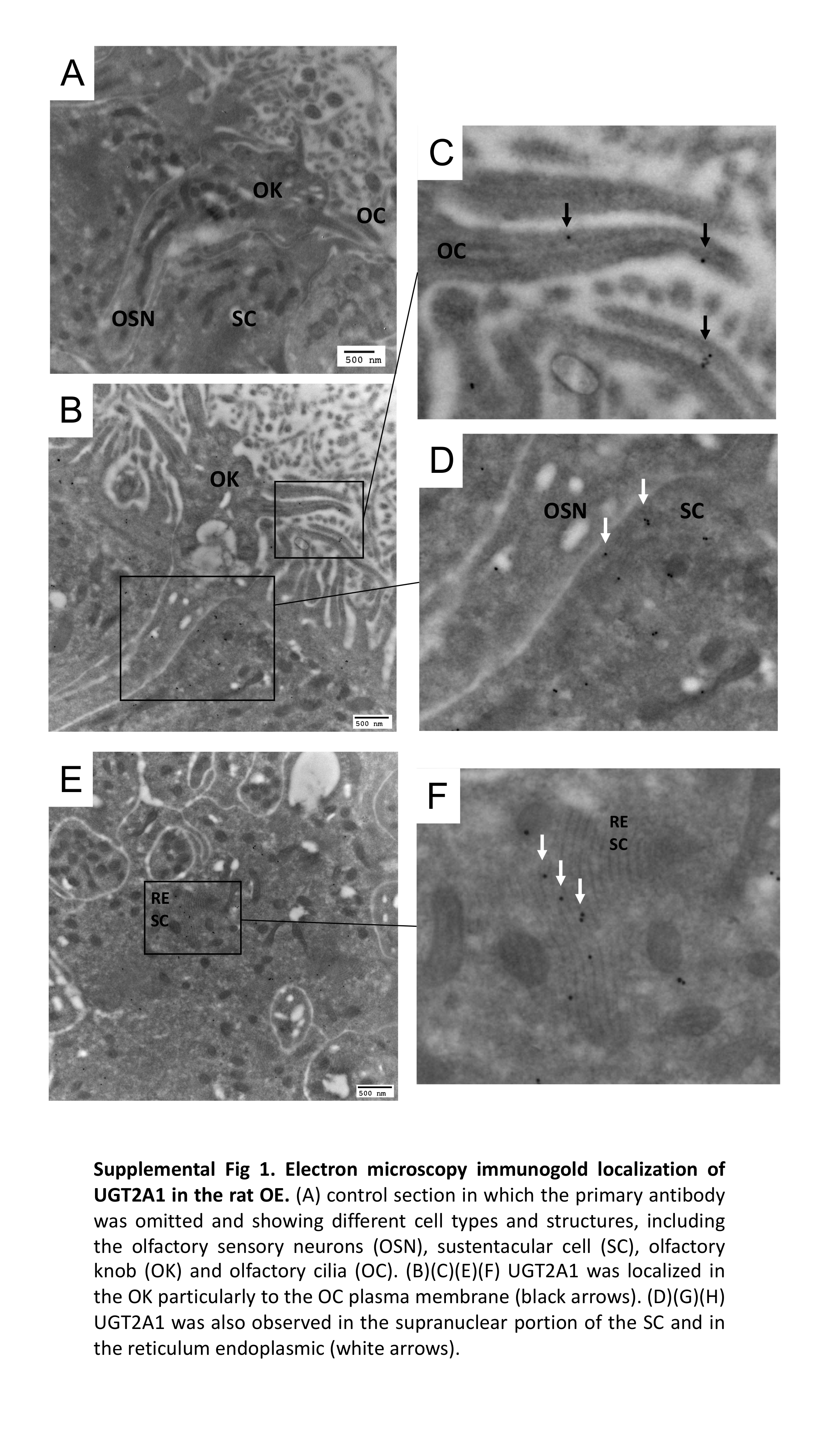

Supplement: S1 Fig — (A) control section in which the primary antibody was omitted and showing different cell types and structures, including the olfactory sensory neurons (OSN), sustentacular cell (SC), olfactory knob (OK) and olfactory cilia (OC). (B)(C)(E)(F) UGT2A1 was localized in the OK particularly to the OC plasma membrane (black arrows). (D)(G)(H) UGT2A1 was also observed in the supranuclear portion of the SC and in the reticulum endoplasmic (white arrows). (TIF) [file pone.0249029.s002.tif]
