# Supplementary material for: The odorant metabolizing enzyme UGT2A1: Immunolocalization and impact of the modulation of its activity on the olfactory response
Source: PLoS One. 2021 Mar 25;16(3):e0249029. doi: 10.1371/journal.pone.0249029 (PMC7993815; doi:10.1371/journal.pone.0249029)
Supplement: S1 Dataset — (PDF) [file pone.0249029.s003.pdf]

Neiers et al. The odorant metabolizing enzyme UGT2A1: Immunolocalization and impact of the modulation of its activity on the olfactory response

Table 1 : ElectroOlfactoGram amplitude data

	Before saline solution		After saline solution				Before Beta-Glucuronidase treatment		After Beta-Glucuronidase treatment			
	Net Amyl Acetate Response Amplitude	Net Eugenol Response Amplitude	Net Amyl Acetate Response Amplitude	Net Eugenol Response Amplitude	After/Before Amyl Acetate Response Ratio	After/Before Eugenol Response Ratio	Net Amyl Acetate Response Amplitude	Net Eugenol Response Amplitude	Net Amyl Acetate Response Amplitude	Net Eugenol Response Amplitude	After/Before Amyl Acetate Response Ratio	After/Before Eugenol Response Ratio
Rat 1	1,05	2,27	1,09	2,35	1,04	1,04	1,03	2,83	0,19	1,44	0,19	0,51
Rat 2	0,75	1,53	0,87	1,68	1,15	1,10	3,78	3,27	2,83	3,90	0,75	1,19
Rat 3	1,27	1,65	1,25	1,44	0,98	0,87	1,83	2,04	2,18	2,56	1,19	1,25
Rat 4	1,26	4,46	0,97	2,57	0,77	0,58	2,73	2,71	2,44	3,49	0,89	1,29
Rat 5	2,49	2,18	1,13	1,35	0,45	0,62	1,04	1,94	0,32	1,82	0,30	0,93
Rat 6	0,71	2,02	1,38	3,06	1,94	1,51	0,72	1,94	0,52	2,60	0,73	1,34
Rat 7	1,94	2,09	0,83	1,18	0,43	0,56	1,65	2,53	2,59	1,32	1,57	0,52
Rat 8	1,74	2,23	2,47	2,38	1,43	1,07	7,29	2,54	3,90	2,94	0,53	1,16
Rat 9	2,91	4,07	1,74	2,80	0,60	0,69	4,21	2,48	4,49	2,69	1,07	1,09
Rat 10	3,69	3,79	2,59	3,66	0,70	0,97	0,78	3,87	1,72	3,70	2,19	0,96
Rat 11	2,38	2,76	0,04	1,60	0,02	0,58	2,74	0,70	1,52	1,12	0,55	1,59
Rat 12	Uncomplete stimulation sequence						0,97	1,15	0,46	1,80	0,47	1,56
Rat 13	2,29	3,98	0,78	1,93	0,34	0,48	3,24	2,33	1,60	2,32	0,49	0,99
Rat 14	1,69	2,73	2,50	4,69	1,48	1,72	0,33	2,36	0,07	3,34	0,22	1,42
Rat 15	Uncomplete stimulation sequence						1,46	0,40	-0,08	1,86	-0,05	4,69
Rat 16	1,42	2,82	0,28	2,80	0,20	0,99	3,10	2,62	3,10	3,59	1,00	1,37
Rat 17	2,58	2,90	2,48	1,42	0,96	0,49	1,04	3,14	0,64	2,20	0,61	0,70
Rat 18	3,73	3,07	3,15	2,06	0,84	0,67	4,10	3,11	3,36	3,44	0,82	1,11
Rat 18	2,18	2,70	1,18	1,69	0,54	0,62	Uncomplete stimulation sequence					
Rat 19	0,78	2,90	2,00	3,24	2,55	1,12	2,06	1,99	3,32	3,52	1,61	1,77
Rat 19	1,97	1,66	0,94	2,11	0,48	1,27	5,57	4,20	2,63	2,91	0,47	0,69

NB : Values in gray cells are discarded outliers.
